# In situ isolation of nuclei or nuclear proteins from adherent cells: a simple, effective method with less cytoplasmic contamination

**DOI:** 10.1186/s40659-023-00429-2

**Published:** 2023-04-21

**Authors:** Ying Qin, Yun Zhou, Kun Wang, Jiaxuan Gu, Zhihao Xiong, Wendiao Zhang, Yong Chen

**Affiliations:** 1 Jiangxi Key Laboratory for Microscale Interdisciplinary Study, Institute for Advanced Study, Nanchang University, Nanchang, 330031 Jiangxi China; 2 College of Life Sciences, Nanchang University, Nanchang, 330031 Jiangxi People’s Republic of China

**Keywords:** Adherent cells, Human umbilical vein endothelial cells (HUVECs), Isolation of nuclei, Lipopolysaccharide (LPS), Nuclear proteins, Western blotting, Triton

## Abstract

**Background:**

Isolation of nuclei or nuclear proteins is a prerequisite for western blot, nuclear proteome profiling, and other evaluations of nuclear proteins. Here, we developed a simple method for in situ isolation of nuclei or nuclear proteins by in situ removing the extranuclear part of adherent cells via a classical nonionic detergent triton X-100.

**Results:**

First, the feasibility of our method was confirmed by confocal microscopy, atomic force microscopy, scanning electron microscopy, dynamic light scattering, immunofluorescence imaging, and time-lapse dynamic observation. Next, the optimal concentration range (approximately 0.1–1% for ~ 10 min) of triton X-100 and the optimal treatment time (< 30 min) of 0.1–1% Triton X-100 for our method were determined via western blotting of eight extra-/intra-nuclear proteins. Subsequently, the effectiveness, sensitivity, and cytoplasmic contamination of our method were tested by investigating the levels of phosphorylated p65 (a NF-κB subunit) in the nuclei of endothelial or tumor cells treated with/without lipopolysaccharide (LPS) via western blotting and by comparing with a commercial nuclear protein extraction kit (a classical detergent-based method). The data show that compared with the commercial kit our method obtained a higher yield of total nuclear proteins, a higher pP65 level in both control and LPS groups, and much lower content of GAPDH (as a reference for cytoplasmic contamination) in nuclei.

**Conclusions:**

The in situ isolation of nuclei or nuclear proteins from adherent cells in this study is a simple, effective method with less cytoplasmic contamination. This method/strategy has the potential of improving the quality of downstream evaluations including western blotting and proteomic profiling.

**Supplementary Information:**

The online version contains supplementary material available at 10.1186/s40659-023-00429-2.

## Background

In many research fields including cell biology, molecular biology, developmental biology, and others, as well as in disease-related research, the determination of signaling pathways and relevant key molecules is vital to understand the underlying molecular mechanisms. External stimuli regulate the expression of specific genes via signaling pathways. The signal can be passed from the extracellular environment to the cytoplasm and then into the nucleus through a series of molecules, inducing significant changes in levels of specific molecules in the nucleus. Therefore, the content measurement of specific molecules in the nucleus is one of the most important aspects in signaling pathway studies.

Western blot is one of widely used methods for evaluating the levels of proteins in cells including those in the nucleus. To evaluate protein level in the nucleus via western blot, the isolation and purification of cell nuclei and total nuclear proteins are required. Moreover, nuclear proteome profiling is very important for accumulating knowledge about regulation of gene expressions and functions. The isolation and purification of cell nuclei and nuclear proteins are also required for nuclear proteome profiling. To date, many methods for isolation of cell nuclei and nuclear proteins have been developed including ultrasonication, acidolysis, enzymolysis, detergent-based extraction, sucrose method, and others [1–7] among which the detergent-based extraction is one of the most widely used methods particularly in commercial nuclear protein extraction Kits.

**Fig. 1:**
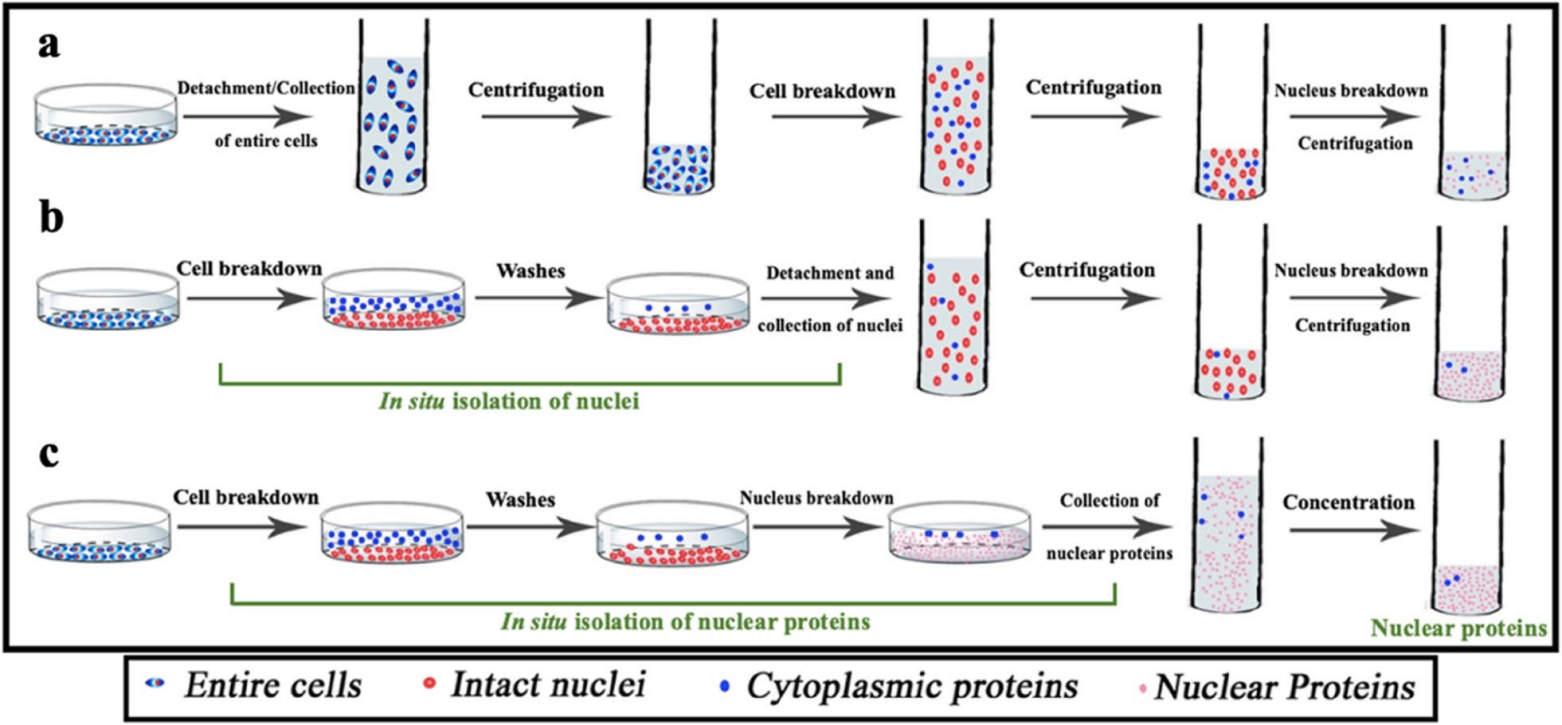
Schematic diagram showing the methods used in this study for in situ isolation of nuclei and nuclear proteins from adherent cells. **a** A classical detergent-based method generally used in commercial nuclear protein extraction kits. **b** Strategy 1 of our method for in situ isolation of nuclei. **c** Strategy 2 of our method for in situ isolation of nuclear proteins

In detergent-based extraction methods, the plasma membrane and cytoplasmic components of cells in suspension are solubilized by a detergent and removed by centrifugation to isolate/purify the nuclei which are further destroyed by another detergent to obtain total nuclear proteins for downstream studies (Fig. 1a). However, the contamination of the isolated nuclei by cytoplasmic debris/proteins in this method is inevitable. Here, a simple, efficient method with less cytoplasmic contamination and higher sensitivity is developed for in situ isolation of the nuclei or nuclear proteins from adherent cells (Fig. 1b,c).

## Materials and methods

### Reagents and cell culture

Triton X-100 and sodium dodecyl sulfate (SDS) were from Solarbio Science & Technology Co. (Beijing, China). Lipopolysaccharide (LPS) was purchased from Sigma. Mouse anti-ICAM-1 mAb and AlexaFluor488-conjugated goat anti-mouse IgG were from Thermofisher Scientific (USA). Monoclonal antibodies (mAb) against phosphorylated P65 (pP65), histone H3 (an internal control for nuclear proteins), and GAPDH (an internal control for cytoplasmic proteins) as well as horseradish peroxidase (HRP)-conjugated goat anti-rabbit IgG were all purchased from Abcam (UK). The rabbit (for western blotting) and mouse (for fluorescence imaging) mAbs against Hif-1a were from Cell Signaling Technology (Boston, MA) and Santacruz (Dallas, TX), respectively. The primary antibody (rabbit polyAb) against low-density lipoprotein (LDLR) and mouse mAb against β-actin were purchased from Proteintech (Wuhan, China). Mouse mAb against lamin A/C (i.e., lamin A + Lamin C) was from Abcam (Cambridge, MA); the second antibodies for immunofluorescence imaging and western blotting were FITC-conjugated goat anti-mouse IgG (Jiangsu ComWin Biotech Co., Ltd., China) and HRP-conjugated goat anti-mouse IgG (Affinity Biosciences, USA), respectively.

Human umbilical vein endothelial cells (HUVECs), purchased from Xiangya Central Experiment Laboratory (Hunan, China) were routinely cultured in RPMI 1640 media (Hyclone) supplemented with 10% (w/v) fetal bovine serum (FBS), 100 U/mL penicillin, and 100 μg/mL streptomycin. Human hepatoma HepG-2 cells, purchased from Cell Bank of Institute of Cellular Biology in Shanghai, Chinese Academy of Science (Shanghai, China), were routinely cultured in DMEM media (BI) supplemented with 10% FBS, 100 U/mL penicillin, and 100 μg/mL streptomycin. Approximately the 5th passage of cells was applied for all experiments.

### In situ imaging of cells treated by Triton X-100 and the nuclei treated by SDS

For the Triton X-100 treatment of cells, HUVECs or HepG-2 cells were plated in a petri dish and grew to reach an approximately 70% confluence. After washing three times with PBS, the cells adhered on substrate of the petri dish were moved onto the sample stage of the LSM710 confocal microscopy. After acquiring an image of cells in a field (this picture was regarded as the 0 time point of treatment), the PBS solution was removed from the petri dish carefully, a solution of Triton X-100 (0.01%, 0.1%, and 1%, respectively in PBS buffer) was added carefully and incubated with the cells for 10 min, and the picture of the cells in the same field was taken again.

For the SDS treatment of cell nuclei, HUVECs were plated in a petri dish and grew to reach an approximately 70% confluence. After washing three times with PBS, the cells adhered on substrate of the petri dish were treated with 0.1% Triton X-100 for 10 min, and gently washed once with PBS. Then, the nuclei adhered on substrate of the petri dish were moved onto the sample stage of the LSM710 confocal microscopy and a picture of cell nuclei in a field was taken (this picture was regarded as the 0 time point of treatment). After removing the PBS carefully, the cell nuclei were incubated with a solution of SDS (0.01%, 0.1%, and 1%, respectively) for a period of time (5, 1, and 0.5 min, respectively), and the picture of the nuclei in the same field was taken again.

### Atomic force microscopy (AFM)

The entire HUVECs and the cells treated with 0.1% Triton X-100 for 10 min were washed three times with PBS, fixed by 4% paraformaldehyde at 37 °C for 30 min, washed twice with PBS, and subjected to an Agilent series 5500 AFM (Agilent Technologies, CA) for imaging in tapping mode under an ambient condition [8]. The data were acquired in PBS using n-silicon tips (PPP-NCH NanoSensors, USA) with an end radius of 10 nm and a force constant of 40 N/m.

### Scanning electron microscopy (SEM)

The HUVECs cells treated with or without 0.1% Triton X-100 for 10 min were washed gently with PBS, fixed by 4% paraformaldehyde at 37 °C for 30 min, washed several times first with PBS and then with double distilled water, and then dehydrated. After the coating of gold by using a sputter coater (Cressington 108 Auto; Cressington Scientific Instruments Ltd., United Kingdom), the samples were subjected to a Quanta 200F Environmental SEM (FEI Co., United Kingdom).

### Dynamic light scattering (DLS)

Three samples for DLS analysis were obtained as follows: HUVECs adhered on the substrate (sample 1: the entire cells in suspension) were treated with 0.1% Triton X-100 for 10 min, rinsed gently three times with PBS, and blew repeatedly with PBS to collect the suspension (sample 2: cell nuclei in suspension); the collected suspension was further treated with 0.1% SDS for ~ 1 min (sample 3: nuclear debris in suspension). The mean sizes of the samples were measured by a dynamic light scattering analyzer (Zetasizer nano zs90, Malvern, UK) as previously reported [9].

### Fluorescence imaging of the effects of Triton X-100 and SDS on cytoplasmic and/or intranuclear contents by confocal microscopy

To test the effects of Triton X-100 on cytoplasmic contents of cells, an organic fluorescent dye named benzocaine-incorporated 1,3-Squaraine (SQ-2), which has the ability of fluorescently staining the cytoplasm of living cells [10], was applied to be a representative of cytoplasmic contents for dynamic observation. To test the effects of Triton and/or SDS on intranuclear contents of cells, the fluorescently stained chromosome and phosphorylated P65 (pP65) were applied as the representatives of intranuclear DNA and protein contents, respectively.

Then, three experiments were designed as follows. In the first experiment, the living HUVEC cells were stained with 10 μg/mL SQ-2 at 37 °C for 30 min and 10 μg/mL hoechst33342 (Beyotime, Shanghai, China) at 37 °C for 15 min; after washing three times with PBS, the cells were put on the sample stage of the LSM710 confocal microscope; after imaging, the solution was replaced with 0.1% Triton X-100 and the cells were imaged at different time points; after around 5 min, the solution was exchanged to 0.1% SDS and the nuclei were imaged again at different time points. In the second experiment, HUVECs were treated with or without 0.1% Triton X-100 for 20 min, gently washed three times with PBS, fixed by 2.5% glutaraldehyde for 20 min, stained with rabbit anti-β-tubulin mAb (1:400; Abcam, Cambridge, MA), Donkey anti-rabbit IgG pAb (1:400; Abcam, Cambridge, MA), and DAPI (Solarbio, Beijing, China) successively (washed with PBS between two steps), and subjected to confocal microscopy. In the third experiment, for HUVECs or HepG-2 cells without depletion of cytoplasmic contents by triton treatment (the control group), the cells were first fixed by 2.5% glutaraldehyde for 20 min, washed three times with PBS, and then treated with 0.1% Triton X-100 for 20 min to promote the cell membrane permeability for immunostaining; for HUVECs or HepG-2 cells with depletion of cytoplasmic contents by Triton treatment, the living cells were first treated with 0.1% Triton X-100 for 10 min and gently washed three times with PBS, and then the remaining nuclei were fixed by 2.5% glutaraldehyde for 20 min. After rinsing three times with PBS, the samples (the intact cells or the remaining nuclei adhered on the substrate) were blocked with 1% bovine serum albumin (BSA) at 37 °C for 1 h, successively stained with ~ 4.75 μg/mL of rabbit anti-pP65 mAb (Abcam, Cambridge, MA) or rabbit anti-Hif-1α mAb at 37 °C for 1 h, ~ 1 μg/mL of AlexaFluor647-conjugated Donkey pAb against rabbit IgG (Abcam, Cambridge, MA) at 37 °C for 1 h, and ~ 5 μg/mL of DAPI (Beyotime, Shanghai, China) at 37 °C for 5 min, respectively (PBS washing was performed in between two steps). After washing with PBS, the samples were imaged by LSM710 confocal microscopy.

### Fluorescence imaging of the effects of Triton X-100 on nuclei membrane and nuclei lamina by confocal microscopy

For the fluorescence staining of membranes, living HUVEC or HepG-2 cells were stained with 5 μM Dio (Jiangsu KeyGEN BioTECH Corp., Ltd., China) at 37 °C for 15 min in the medium supplemented without FBS, washed, incubated twice with the complete medium at 37 °C for 10 min, washed with PBS, and subjected to confocal microscopy. The fluorescence staining of nuclear lamina was similar to that of nuclear pP65. The primary and second antibodies were mouse mAb against lamin A + Lamin C (1:1000; Abcam, Cambridge, MA) and FITC-conjugated goat anti-mouse IgG (1:1000; Jiangsu ComWin Biotech Co., Ltd., China), respectively.

### Detection of ICAM-1 expression at protein and mRNA levels

For the fluorescence detection of ICAM-1 expression on cell surfaces, the cells were treated with 0, 0.1, 0.5, 1 μg/mL LPS for 12 h at 37 °C in a CO_2_ incubator, and washed twice with PBS, fixed with 4% paraformaldehyde at 37 °C for 20 min, washed three times with PBS, blocked with 2% bovine serum albumin (BSA) at 37 °C for 1 h, incubated with anti-ICAM-1 primary antibody solution containing 2% BSA at 37 °C for 1 h, washed three times with PBS, and incubated with AlexaFluor488-conjugated 2nd antibody solution containing 2% BSA at 37 °C for 1 h. After washing three times with PBS, the samples were subjected to An LSM710 confocal microscopy (Carl Zeiss, Oberkochen, Germany) equipped with a Zeiss inverted microscope and a Zeiss Plan-Neofluar objective (40 × /0.75) for fluorescence imaging.

For the detection of ICAM-1 expression at mRNA level via RT-PCR, after cells were treated with LPS at different concentrations and washed with PBS, the total RNA was extracted via the E.Z.N.Z. Total RNA Kit (OMEGA, USA) according to the manufacturer’s introduction. The HiFiScript cDNA Synthesis Kit (ComWin Biotech Co., Ltd., Beijing, China) was used for the synthesis and amplification of cDNA on ice according to the manufacturer’s introduction. The used primer sequences for *ICAM-1* have been reported in our previous studies [11, 12]. The forward and reverse primers for *β-actin* (an internal reference) are 5′-AGCGAGCATCCCCCAAAGTT-3′ and GGGCACGAAGGCTCATCATT-3′, respectively. The DNA lengths of *ICAM-1* and *β-actin* as an internal reference are 556 bp and 285 bp, respectively. The DNA was detected by agarose gel electrophoresis on a 0.8% agarose gel at 110 V for 30 min and imaged using a gel imaging system (GelDoc 2000, Bio-Rad, CA).

### Isolation of cell nuclei and total nuclear proteins

The same batch of HUVEC or HepG-2 cells was divided in quadruplicate with equal volume, two for the commercial kit method (one for the control group and the other one for the LPS group) and the other two for our method (the control and LPS groups, respectively). The cells were planted in flasks and grew for ~ 24 h. After washing with PBS, the cells were treated without (the controls) or with 1 μg/mL LPS (the LPS groups) for 12 h in the CO_2_ incubator.

For the isolation of cell nuclei and total nuclear proteins by our method, the following procedure was performed for both control and LPS groups. After gently washing three times with PBS, the cells adhered on the substrate were incubated with 500 μL of 0.1% pre-cooled Triton X-100 solution in PBS buffer (NaCl, Na_2_HPO_4_･12H_2_O, NaH_2_PO_4_･2H_2_O; 0.01 M in double distilled water) supplemented with a proteinase inhibitor cocktail including phenylmethylsulfonyl fluoride (PMSF), basic proteinase inhibitor, and phosphatase inhibitor for 10 min on ice. After carefully removing the solution and washing three times (tilting the flasks and letting the solution flow down) with the Triton X-100 solution supplemented with the protein inhibitor cocktail, the nuclei adhered on the substrate were collected by a physical method (e.g. blowing the substrate with the solution repeatedly) into pre-cooled centrifuge tubes. After spinning at 1000×*g* for 10 min at 4 °C, the pellets containing cell nuclei were incubated with 0.1% pre-cooled SDS solution supplemented with the proteinase inhibitor cocktail by vortexing three times (each for 15–20 s with an interval of 10 min) on ice. After spinning at 13,000×*g* for 20 min at 4 °C, the supernatants containing total nuclear proteins were collected for protein concentration assay and western blotting.

For the isolation of cell nuclei and total nuclear proteins using the commercial kit, the experiment was performed both control and LPS groups according to the product manual of the nuclear/cytoplasmic protein extraction Kit (Cat. #: KGP150; Jiangsu Keygen Biotech Corp., Ltd., Nanjing, China). The cells treated with or without LPS were washed three times with PBS and detached from the substrate by trypsin digestion method. After the cells in suspension were span at 800×*g* for 3 min at 4 °C, the pellets were washed two times with pre-cooled PBS, and incubated with 450 μL pre-cooled buffer A containing the proteinase inhibitor cocktail and 50 μL buffer B for 30 min on ice. After spinning at 3000 rpm for 10 min at 4 °C, the pellets were incubated with 100 μL pre-cooled buffer C containing the proteinase inhibitor cocktail by vortexing three times (each for 15–20 s with an interval of 10 min) on ice. After spinning at 14,000×*g* for 30 min at 4 °C, the supernatants containing total nuclear proteins were collected for protein concentration assay and western blotting.

### BCA protein assay and western blot

The BCA protein assay and western blot were performed as usual. Briefly, the concentrations of the total nuclear proteins were quantified by a commercial BCA protein assay kit (ComWin Biotech Co., Ltd., Beijing, China) according to the product manual and detected at 562 nm. For western blot, the nuclear protein samples in loading buffer (~ 20 μL at a protein concentration of 1 μg/μL) were separated on a SDS-PAGE gel made up of a 10% separating gel and a 5% concentrating gel, transferred to nitrocellulose (NC) membranes at 4 °C, blocked with 5% BSA-containing TBST buffer, immune-stained by the primary and HRP-conjugated secondary monoclonal antibodies against pP65, histone H3, and GAPDH, and chemiluminescently detected as reported in our previous study [12].

### Statistical analysis

All data from at least three independent experiments were expressed as mean ± SD in the text. Student’s *t*-test was performed. A significant difference was achieved when *p* < 0.05.

## Results and discussion

In the classical detergent-based extraction method (Fig. 1a), the adherent cells were detached from the substrate and treated in suspension by a detergent, followed by a centrifugation to remove the cytoplasmic debris and to harvest the nuclei. However, many cytoplasmic debris may remain in the nuclei pellets even due to weak interactions (e.g. electrostatic interaction) causing sample contamination. If the cells are treated by the detergent under an adhesion condition after which the nuclei remain under the adhesion condition, the cytoplasmic debris including those electrostatically associated with the nuclei can be easily removed by washing from the nuclei adhered on the substrate. The in situ isolation of nuclei probably helps to obtain purer nuclei with less cytoplasmic contamination, finally resulting in a more sensitive measurement (Fig. 1b). Moreover, the adherent nuclei can be further disrupted in situ to directly obtain the total nuclear proteins (the process is showed in Fig. 1c).

**Fig. 2:**
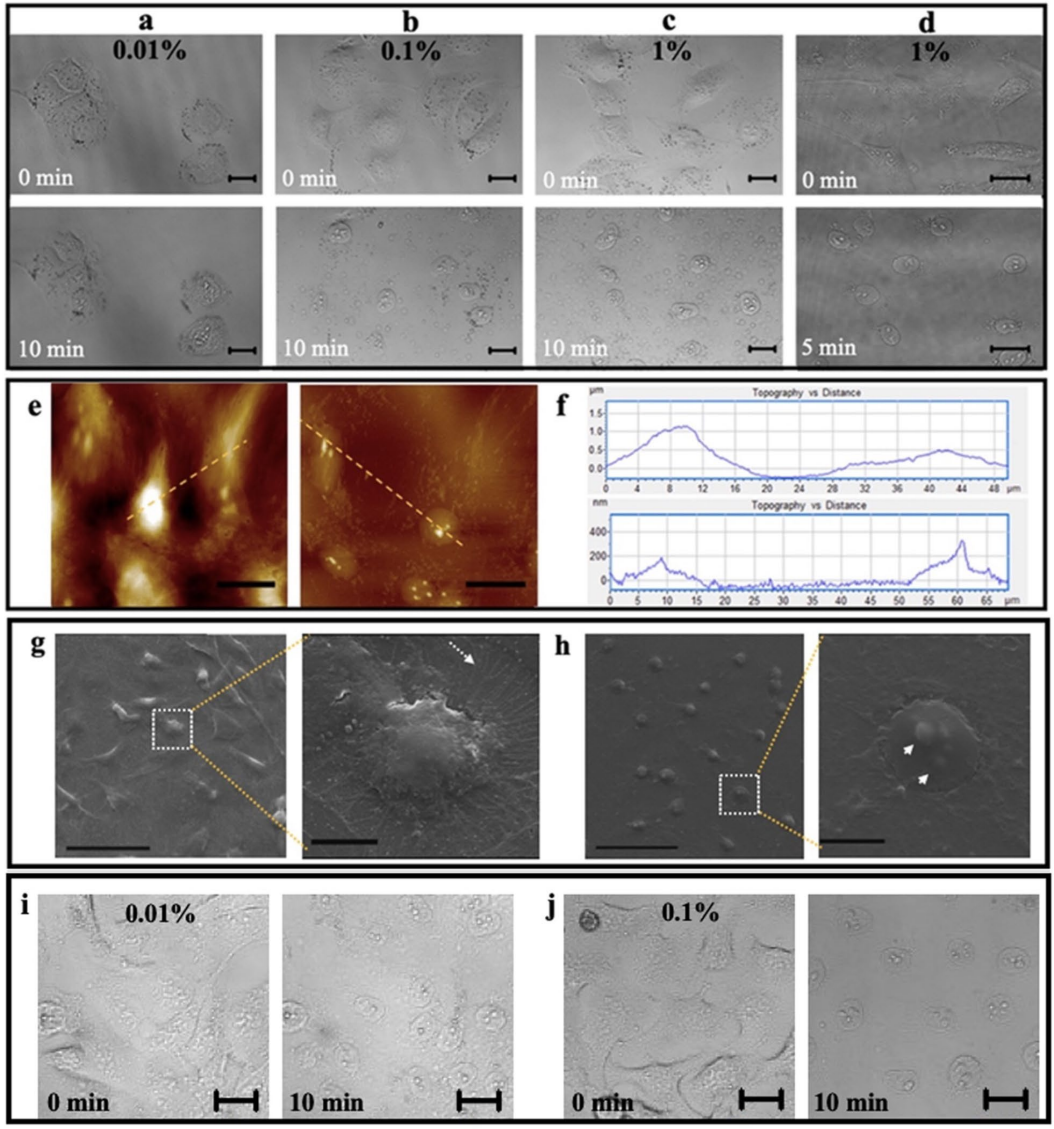
Morphological validation of the in situ isolation of nuclei from adherent cells. **a–d** In situ confocal microscopic observation of HUVECs treated with 0.01%, 0.1%, and 1% Triton X-100, respectively for 10 min without subsequent PBS washes and with 1% Triton X-100 for 5 min with subsequent PBS washes. Upper panels: before treatment; bottom panels: after treatment. **e** AFM imaging of HUVECs treated without (left) and with (right) 0.1% Triton X-100 for 10 min. **f** The height profiles of the cross sections along the lines in AFM images. Upper and bottom panels are corresponding to the left and right panels, respectively in the AFM images. **g** SEM imaging of HUVECs without treatment. The white arrow indicates the filamentous cytoskeleton in the cytoplasm. **h** SEM imaging of HUVECs after 0.1% Triton X-100 treatment for 10 min. The white arrowheads indicate the nucleoli inside the nucleus. **i**, **j** In situ confocal microscopic observation of HepG-2 cells treated with 0.01% (**i**) and 0.1% (**j**) Triton X-100, respectively for 10 min. Left panels: before treatment; right panels: after treatment. Scale bars: (**a–e**, **i**, **j**) 20 μm; (**g** and **h**) left panel: 50 μm; right panel: 10 μm

To test the possibility that detergent can deplete the cytoplasmic component of adherent cells and keep the nuclear component intact and adherent on the substrate, the in situ morphological changes of the same HUVEC cells before and after detergent treatment (Triton X-100, a widely used nonionic detergent/surfactant for cell breakdown) were observed by confocal microscopy (Fig. 2a–d). It seems that 0.01% Triton X-100 treatment for 10 min induced no changes in cellular morphology (Fig. 2a). On the contrary, the treatments of Triton X-100 at 0.1% (Fig. 2b) and 1% (Fig. 2c) for 10 min caused the disappearance of the cytoplasmic component of the cells, the appearance of many particles in suspension which were mainly the cytoplasmic debris, and the maintaining of the intact nuclei at the original position. After carefully washing with PBS, the suspended cytoplasmic debris/proteins could be removed and the clean, intact nuclei could be left at the original position (Fig. 2d). The topographical images (Fig. 2e) and the corresponding cross-sectional height profiles (Fig. 2f) of the adherent cells treated with or without detergent treatment (0.1% Triton X-100 for 10 min) detected by atomic force microscopy (AFM) further confirmed the result, as well as by scanning electron microscopy (SEM; Fig. 2g, h). Before treatment, the cytoplasm (left panel of Fig. 2g) and the filamentous cytoskeleton in the cytoplasm (indicated by the white arrow in the right panel of Fig. 2g) were observed by SEM; after 0.1% Triton X-100 treatment, the cytoplasm (left panel of Fig. 2h) and the cytoskeletal filaments (right panel of Fig. 2h) disappeared whereas the SEM observed the nucleoli beneath the nuclear membrane (indicated by the white arrowheads in the right panel of Fig. 2h) which were undetectable before Triton treatment due to the existence of the plasma membrane and the cytoplasm. Moreover, a smooth outer surface of the in situ isolated nucleus, instead of a network-like structure (i.e., the lamina underneath the nuclear membrane), was imaged (right panel of Fig. 2h) implying that the nuclear membrane was not damaged by Triton X-100. Similar results on the Triton X-100-induced morphological changes of HepG-2 cells (a type of cancer cell) also were observed by in situ confocal imaging (Fig. 2i, j). We also tested the effects of other detergents (e.g. digitonin and saponin) on living cells. However, both digitonin (Additional file 1: Fig. S1) and saponin [13] could not completely deplete the cytoplasmic contents of cells even at a relatively high concentration of detergent (e.g. 1%).

**Fig. 3:**
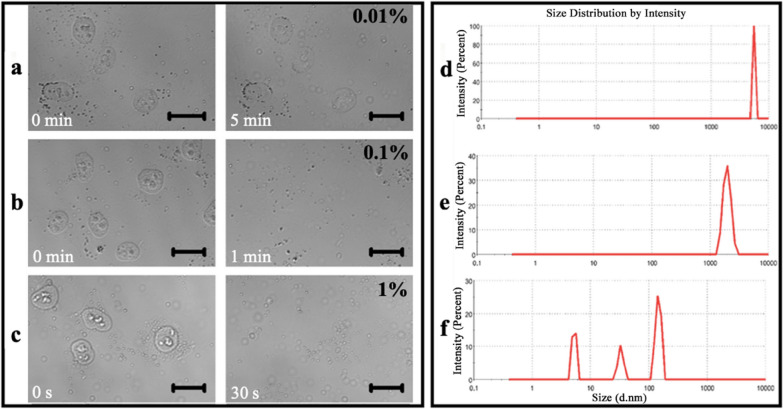
Morphological and size validation of the in situ isolation of nuclear proteins from adherent nuclei. **a**–**c** In situ confocal microscopic observation of cell nuclei treated with 0.01%, 0.1%, and 1% SDS, respectively without PBS washes. The nuclei were obtained from HUVECs pre-treated with 1% Triton X-100 for 10 min. Left panels: before treatments; right panels: 5 min, 1 min, and 30 s, respectively after treatments. Scale bars: 20 μm. **d**–**f** Size distributions of the entire cells, the intact nuclei after Triton X-100 treatment, and the nuclear debris/proteins after SDS treatment, respectively detected by DLS

Next, the in situ morphological changes of the adherent nuclei before and after detergent treatment (SDS, a widely used anionic detergent/surfactant for cell lysis and nucleus breakdown) were also observed by confocal microscopy (Fig. 3a–c) to test the possibility that detergent can break down the nuclei and release nuclear proteins in situ. Similarly, the nuclei remained intact after treating with 0.01% SDS for 5 min (Fig. 3a) whereas 0.1% and 1% SDS could induce the disappearance of the nuclei and the appearance of many suspended particles (i.e. the nuclear debris/proteins) within 1 min (Fig. 3b) and 30 s (Fig. 3c), respectively.

To further confirm the results, the samples from the abovementioned three steps including the entire cells, the intact nuclei (i.e. the remaining of the cells after 0.1% Triton X-100 treatment for 10 min), and the nuclear debris (i.e. the final solution after the nuclei were treated with 0.1% SDS for ~ 1 min) were detected by dynamic light scattering (DLS). The size distribution displays a gradual decrease from entire cells (Fig. 3d) to the intact nuclei (Fig. 3e) and to the nuclear debris (Fig. 3f) with an average size of 6.85 μm, 2.25 μm, and 130.6 nm (the average of three peaks), respectively. Although this method (DLS) is not very accurate for the measurement of relatively large particles (e.g. at the micrometer scale), the results still can reflect the reality and confirm the effects of the detergents (Triton X-100 and SDS) on the cells and the nuclei.

**Fig. 4:**
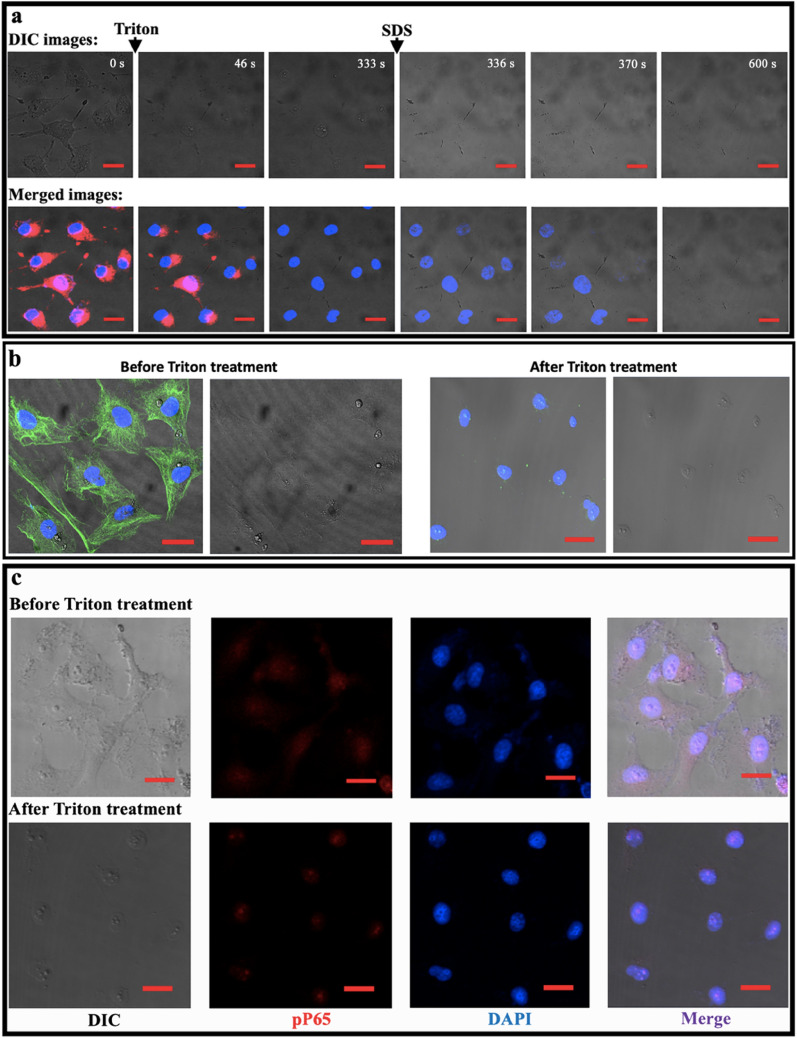

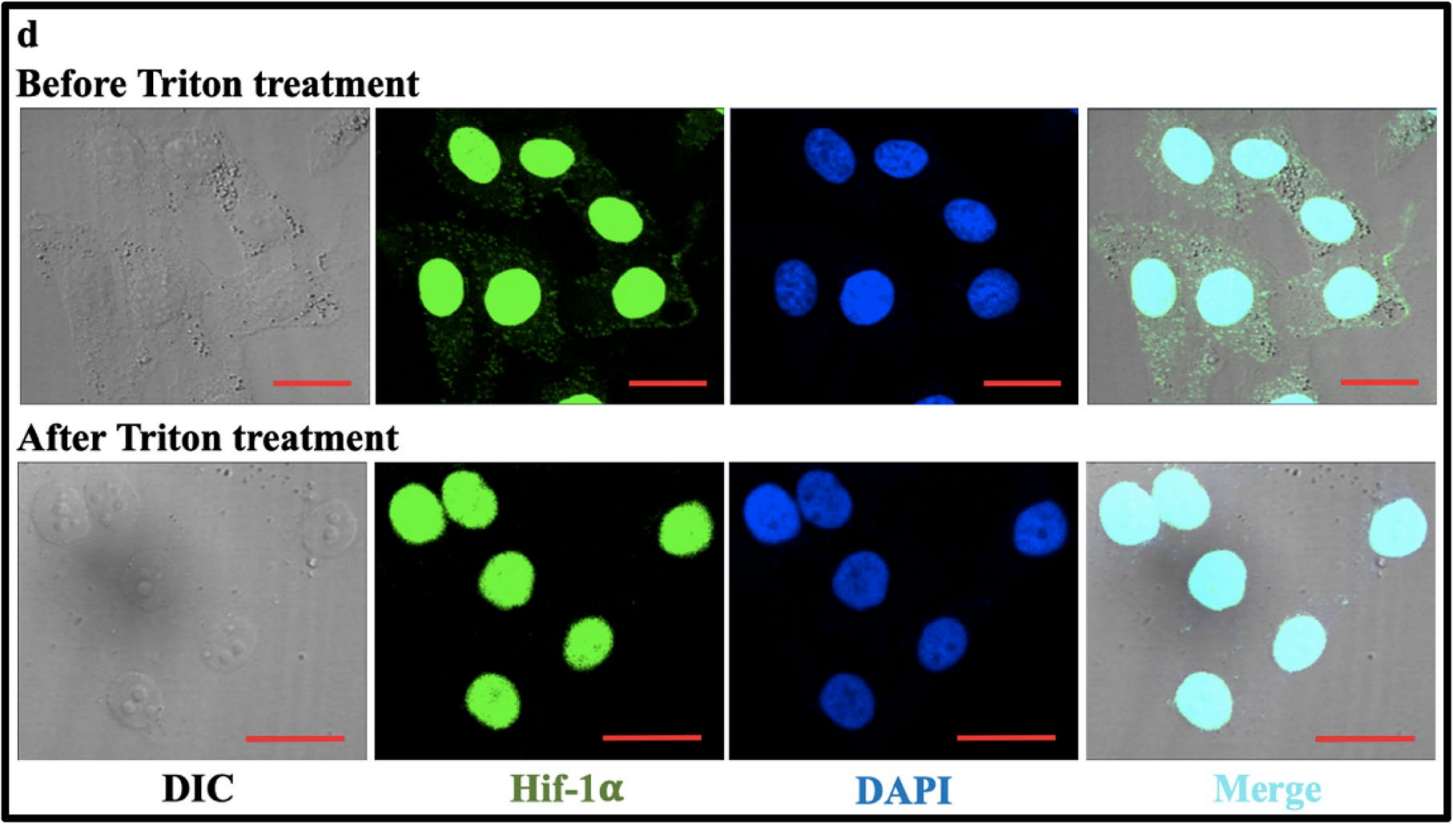
Fluorescence confocal imaging visualizes the effects of the in situ isolation of nuclear proteins from adherent nuclei on the cytoplasmic and intranuclear contents of HUVEC cells. **a** Dynamic observation of the morphological and fluorescence changes of cells after successive Triton X-100 (0.1%) and SDS (0.1%) treatments as indicated by the black arrows. Upper panels: DIC images (the imaging focus is on cell morphology); lower panels: images merged from DIC and fluorescence images (the imaging focus is on fluorescence). The cytoplasmic contents were stained with a red fluorescent dye (SQ-2) and the intranuclear chromosomes were stained with Hoechst33342 (blue). **b** Depleting effect of 0.1% Triton X-100 for 10 min on tubulin cytoskeleton. Left panels: images merged from DIC and fluorescence images (tubulin cytoskeleton in green and the nuclei in blue); right panels: DIC images. **c**, **d** No effects of 0.1% Triton X-100 on intranuclear contents including chromosomes and proteins (e.g., pP65 in **c** and Hif-1α in **d** as representatives). The HUVEC cells have been activated by 1 μg/mL LPS prior to Triton treatment. The chromosomes were stained with DAPI (blue), pP65 was stained with anti-pP65 antibody and AlexaFluor647-conjugated anti-IgG antibody (red), and Hif-1α was stained with anti-Hif-1α mAb and FITC-conjugated goat anti-mouse IgG. Scale bars: 20 μm (**a**–**d**). Additional file 2: Video S1 also shows the time-lapse observation of the effects of successive Triton X-100 and SDS treatments on the mitochondria and nuclei of cells fluorescently stained with Mitotracker (red) and Hoechst33342 (blue)

Then, we utilized fluorescence imaging to further confirm the above results. In the time-lapse dynamic observation (Fig. 4a), 0.1% Triton X-100 treatment caused the gradual disappearance of Squaraine (SQ-2) fluorescent dye molecules in cytoplasmic compartments of living HUVEC cells and kept the Hoechst33342-stained nuclei unchanged whereas 0.1% SDS treatment induced the gradual nuclear vanishment in both morphology (upper panels) and fluorescence (lower panels). The data implies that Triton treatment can deplete the cytoplasmic contents but without influence on the intranuclear contents [14] and that SDS treatment can break down the nuclei. Figure 4b shows that after Triton X-100 treatment the cytoskeleton (microtubule immunostained in green) in cytoplasm disappeared while the nuclei (stained with DAPI in blue) remained unchanged [14], further confirming that Triton treatment can deplete the cytoplasmic contents. Figure 4c, d shows the immunofluorescence staining of phosphorylated P65 (pP65) and hypoxia inducible factor Hif-1α (both are mainly located in the nucleus) in intact LPS-activated HUVEC cells (upper panels) and in the remaining nuclei after Triton treatment (lower panels). The maintenance of the fluorescent dyes specifically labeled on the intranuclear molecules (i.e., pP65, Hif-1α, and DNA) in the remaining nuclei after Triton treatment further verified that Triton X-100 treatment did not cause the leak of intranuclear molecules (e.g. proteins and DNA) in the present study. Similar results about pP65 were also observed in Triton-treated LPS-activated HepG-2 cells (Additional file 1: Fig. S2).

To obtain some phenomena which are unable to be observed by the stationary snapshots, a time-lapse Additional file 2: Video S1 is utilized to display the Triton/SDS-induced dynamic changes in different parts of HUVEC cells which were fluorescently stained for mitochondria and nuclei with mitotracker (red) and Hoechst33342 (blue), respectively. Before treatments, the suspension is clear and the fluorescently stained mitochondria (red) were confined inside the clear boundary of cells with fluorescently stained nuclei (blue). After 0.1% Triton addition, the cell boundary quickly became hazy; many particles were released and appeared in suspension among which some probably were membrane vesicles derived from the plasma membrane; the red fluorescence (mitochondria) gradually disappeared; and mainly nuclei still remained on the substrate. These phenomena imply the breakdown of the plasma membrane and the release of cytoplasmic contents including the fluorescently stained mitochondria. On the other hand, during the whole process of Triton treatment different parts of cell nuclei remained unchanged (e.g., the nuclear boundary/membrane, the particles inside the nuclei, and the blue fluorescence), implying that the cell nuclei were not disturbed by Triton. However, after 0.2% SDS treatment, the nuclear boundary rapidly blew up, the particles inside nuclei spread outward, and the blue area became larger and larger and finally disappeared. These phenomena provide direct evidence supporting that SDS treatment caused the breakdown of nuclear membrane and the release of the nuclear contents.

**Fig. 5:**
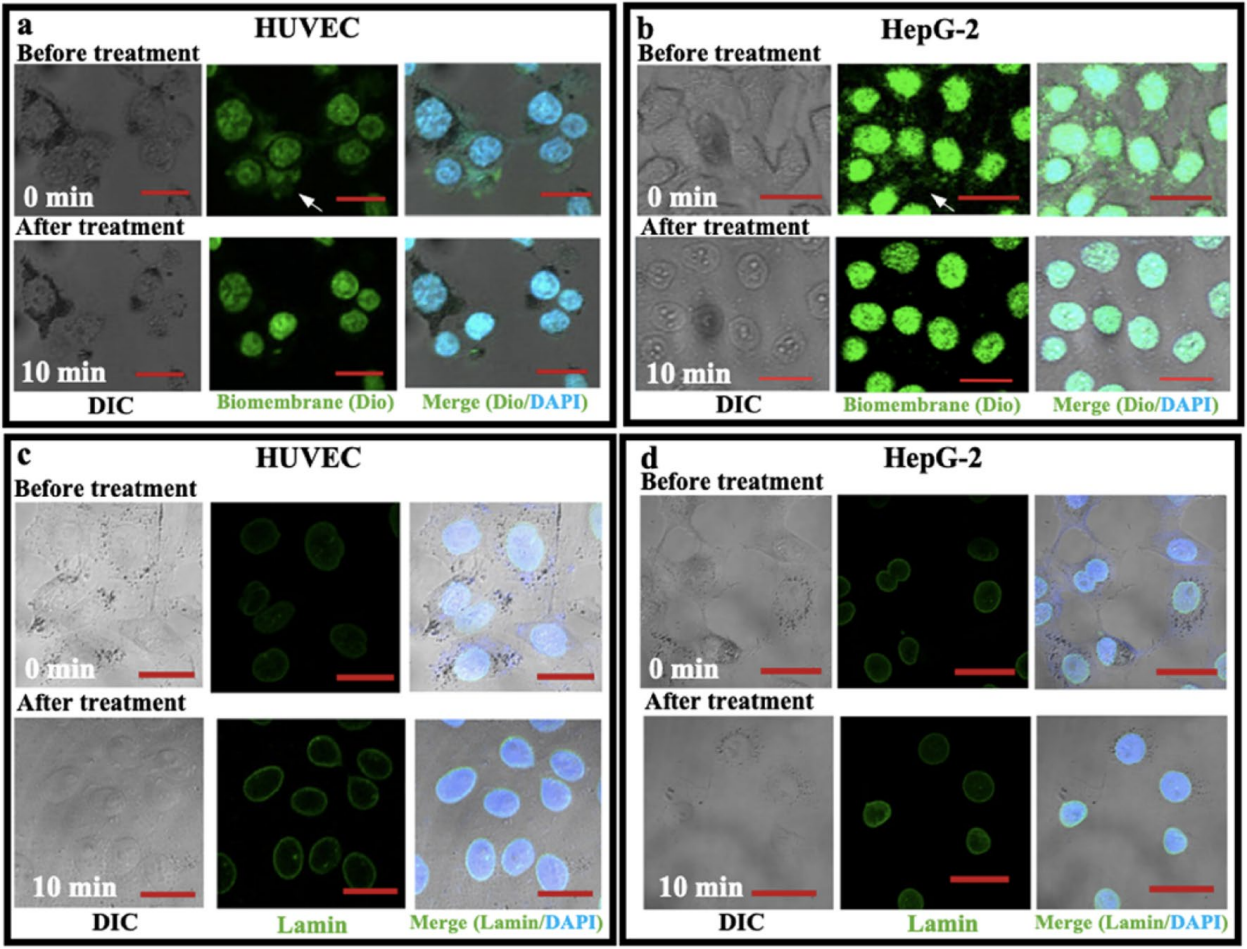
In situ confocal observation of the effects of 0.1% Triton X-100 treatment for 10 min on nuclear membrane and nuclear lamina. **a**, **b** Effects on nuclear membranes of HUVEC (**a**) and HepG-2 (**b**) cells stained with Dio (a fluorescent dye for lipids). **c**, **d** Effects on nuclear Lamina (a nuclear cytoskeleton beneath nuclear membrane) of HUVEC (**c**) and HepG-2 (**d**) cells stained with anti-Lamin (Lamin A + Lamin C) mAb and FITC-conjugated 2nd antibody. Upper panels: before treatment; bottom panels: 0.1% Triton X-100 treatment for 10 min. Left panels: DIC images; middle panels: fluorescent images of biomembranes (**a**, **b**) or lamina (**c**, **d**) in green; right panels: images merged from DIC and fluorescence images including DAPI in blue. The arrows in the upper panels of **a** and **b** indicate the extranuclear membranes stained by Dio which disappears after Triton treatment (the bottom panels of **a** and **b**). Scale bars: 20 μm (**a**–**d**)

To further test whether Triton treatment destroys the envelop of cell nucleus, nuclear membrane and nuclear lamina, two major components of a nuclear envelop, were fluorescently stained by Dio for lipids/biomembranes and anti-lamin mAb for lamina, respectively, and in situ imaged (Fig. 5). Dio, a fluorescent dye for lipids, is able to stain biomembranes including the nuclear membrane. Without Triton treatment, both the nuclear membrane and extranuclear membranes (as indicated by the arrows in the upper, middle panels of Fig. 5a, b) were fluorescently stained in green; upon treatment, the extranuclear fluorescence disappeared whereas the fluorescence in nuclear area remained (Fig. 5a, b). It implies that Triton X-100 could not destroy the nuclear membrane at present condition. Figure 5c, d shows that Triton treatment was also unable to destroy the nuclear lamina. These data, in combination with the morphological data and the fluorescence data about pP65 and DNA/chromosome (DAPI), imply that Triton treatment at the condition in this study is unable to break down the envelop of cell nucleus which prevents nuclear contents/proteins from leaking out of the nucleus.

All data above (Figs. 2, 3, 4, 5) prove the feasibility of in situ isolation of nuclei or nuclear proteins from adherent cells. During in situ isolation of nuclei, a few cell-bound membrane vesicles (CBMVs) might remain on the substrate (e.g., the small particles in the bottom image of Fig. 2d). These vesicles probably were partially responsible for the peak with an average size of more than 100 nm (Fig. 3f). Although in a relatively small quantity, cell-bound membrane vesicles (particularly the vesicles with small sizes) might be a source of contamination of nuclear proteins for our method and previous methods due to their property of resistance to various detergents including Triton X-100 and SDS [13, 15] and to the containing of some nuclear proteins [16]. After Triton treatment, the remaining nuclei on the substrate will be gently washed to remove the cytoplasmic debris prior to the collection of isolated nuclei. Careful, gentle washes will not significantly detach the attached nuclei from the substrate (Additional file 1: Fig. S3) [15], but violent washing must be avoided.

**Fig. 6:**
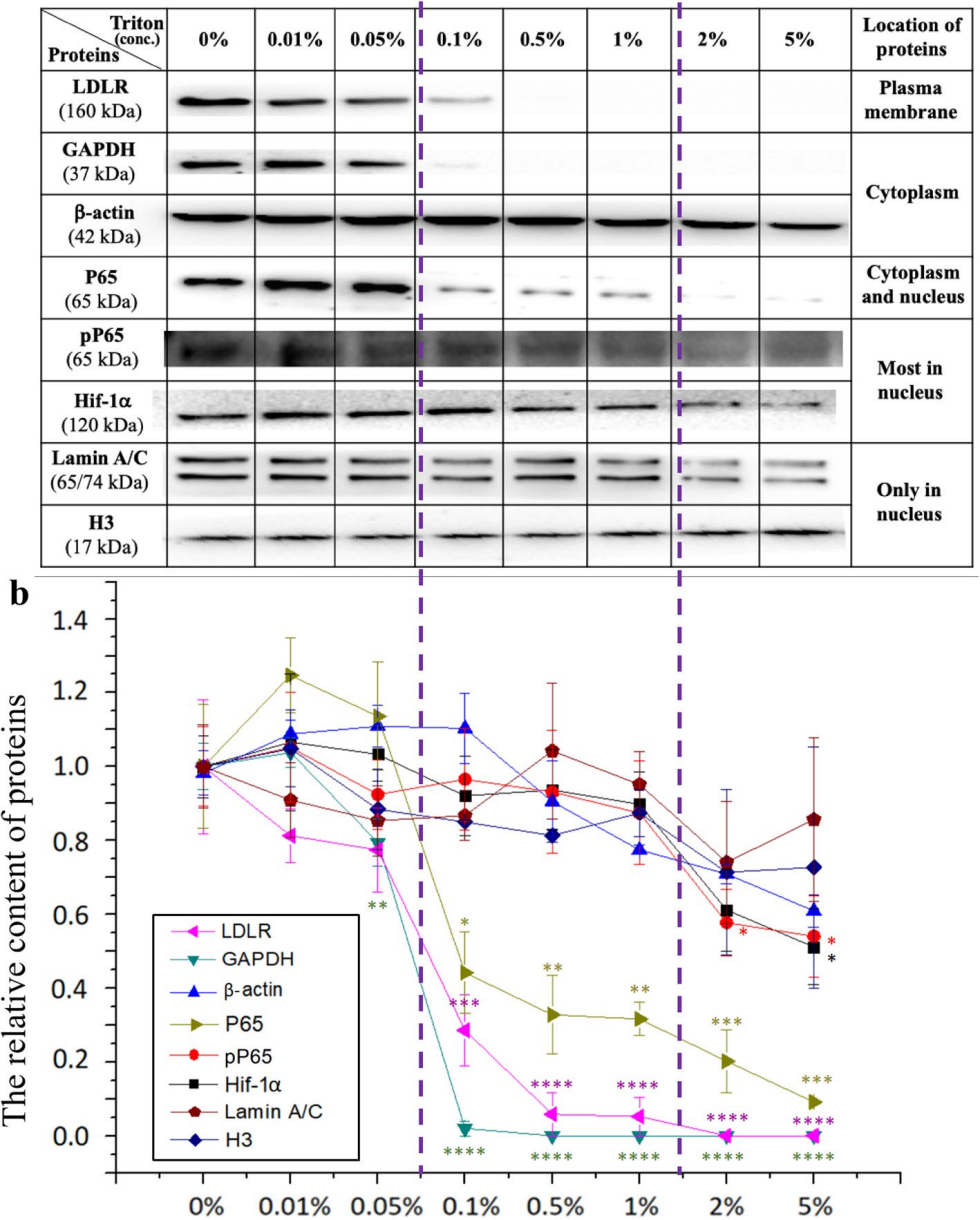
Effects of triton treatment at different concentrations on extra- and intra-nuclear proteins detected by western blotting. **a** Representative western blot data of different extra-/intra-nuclear proteins (LDLR, GAPDH, β-actin, P65, pP65, Hif-1α, Lamin A/C, and H3, respectively at different cellular locations as indicated in the table) of the HUVEC cells treated by Triton X-100 at different concentrations (0, 0.01%, 0.05%, 0.1%, 0.5%, 1%, 2%, and 5%, respectively) for 10 min. **b** Quantitative analysis of the western blot data from three independent experiments (mean ± SEM). *p < 0.05; **p < 0.01; ***p < 0.001; ****p < 0.0001 compared with the control (the 0% group). The cells for pP65/Hif-1α detection were pre-activated by 1 μg/mL LPS. LDLR: Low-density lipoprotein receptor; GAPDH: Glyceraldehyde-3-phosphate dehydrogenase; P65: A subunit of transcription factor NF-κB; pP65: phosphorylated P65; Hif-1α: The α subunit of hypoxia inducible factor-1; H3: Histone H3

Subsequently, the optimal concentration range of triton X-100 in our strategies were investigated by using different concentrations of triton X-100 (0%, 0.01%, 0.05%, 0.1%, 0.5%, 1%, 2%, and 5%, respectively for 10 min). Eight proteins including one plasma membrane protein (low-density lipoprotein receptor or LDLR) two cytoplasm proteins (GAPDH and β-actin), one protein located in both cytosol and in the nucleus (P65 which actually contains pP65), and four intranuclear proteins (pP65, Hif-1α, lamin A/C, and histone H3) were tested via western blotting (Fig. 6). Both the representative western bolt data (Fig. 6a) and the quantitative analysis (Fig. 6b) show the following results: (a) When the concentration of triton was ≤ 0.05%, a relatively high levels of all proteins were detected, implying that triton did not damage the plasma membrane and that no intracellular proteins were lost; (b) when ≥ 0.1% triton was applied, the concentrations of the two extranuclear proteins (i.e., LDLR and GAPDH) dramatically (*p* < 0.001) dropped to a very low level, and the concentration of P65 also significantly (*p* < 0.05) decreased, implying that the cell boundary has been destroyed by triton and that all extranuclear proteins including the extranuclear P65 were released (the intranuclear P65 (i.e., pP65) still remained); (c) when the triton concentration reached ≥ 2%, the concentration of P65 further decreased to a much lower level probably because triton at a relatively high concentration also damaged the nuclear membrane losing the intranuclear P65 molecules (i.e., pP65), at the same time the relative content of Hif-1α dropped significantly; (d) up to 5% triton could not cause a statistically significant decrease in the content of lamin A/C and H3, implying that many proteins associated with chromosomes or nuclear skeleton can be retained even under the condition of no nuclear membrane; (e) surprisingly but interestingly, the relative content of β-actin (a cytoskeletal protein in cytosol) also has no statistically significant change after Triton treatment while another cytoskeletal protein (β-tubulin) could be thoroughly removed by 0.1% Triton (Fig. 4b), implying that β-actin was probably responsible for the remaining of nuclei on substrates. Therefore, the data show that approximately 0.1%-1% is the optimal concentration range of triton treatment for ~ 10 min in our strategies. Triton of < 0.1% for 10 min cannot remove many cytoplasmic proteins while triton of ≥ 2% may cause the loss of some intranuclear free proteins. However, it is possible that a change in triton treatment time may induce a little shift of this optimal concentration range.

**Fig. 7:**
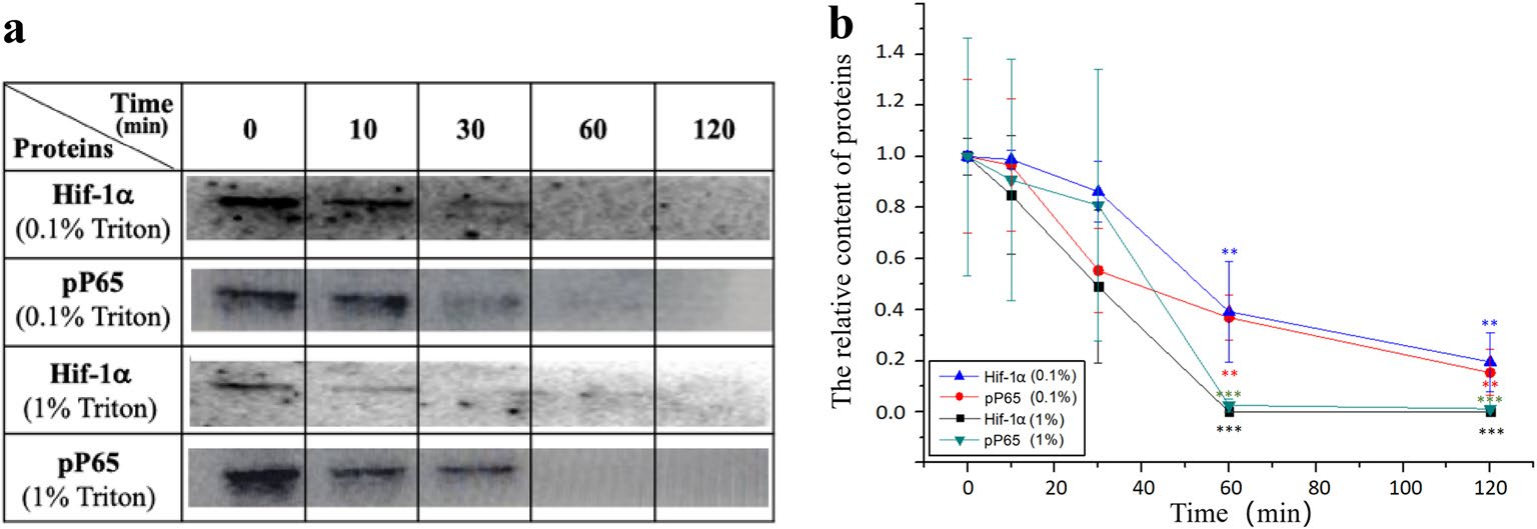
Effects of treatment time of 0.1% or 1% triton on the contents of intranuclear proteins (Hif-1α and pP65) detected by western blotting. **a** Representative western blot data of two intranuclear proteins (i.e., Hif-1α and pP65) of the HUVEC cells, which were pre-activated by 1 μg/mL lipopolysaccharide (LPS), treated by 0.1% or 1% Triton X-100 for various time periods (0, 10, 30, 60, and 120 min, respectively). **b** Quantitative analysis of the western blot data from three independent experiments (mean ± SEM). **p < 0.01; *** and *p* < 0.001; **** compared with the control (i.e., the 0 min group without Triton treatment in which group the total proteins of Hif-1α/pP65, instead of only intranuclear proteins, were harvested for western blotting). Hif-1α: the α subunit of hypoxia inducible factor-1; pP65: phosphorylated P65

Then, we evaluated the effect of Triton treatment time on the isolation of nuclei by using two intranuclear proteins (Hif-1α and pP65) via our method (Fig. 7). We found that Triton treatment at both 0.1% and 1% for less than 30 min could not induce a significant decrease in relative content of both proteins while treatment for ≥ 60 min caused a decrease of more than 50% in protein content. Therefore, it seems that < 30 min is optimal for 0.1–1% Triton treatment in the current study. It is possible that a Triton treatment at a relatively lower concentration (e.g., < 0.1%) for a longer time period (e.g., > 30 min) is also optimal for our method. Moreover, the optimal conditions may be different for different cells types. We did not test these possibilities in the current study. Considering the possibility of protein loss due to the non-specific sticking to glassware, we also compared the yield of nuclear proteins (Hif-1α and pP65) isolated by our method with the yield from total cell protein isolated by RIPA (i.e., nuclear vs. total Hif-1α/pP65). No significant differences were detected (Additional file 1: Fig. S4) excluding the possibility of protein loss due to non-specific sticking.

To further test the effectiveness and sensitivity of our method, the content of the phosphorylated form of P65 (a subunit of NF-κB, a transcription factor and a key molecule in multiple signaling pathways) in the nuclei of the cells treated with or without lipopolysaccharide (LPS) was measured via western blot. It has been reported that LPS could promote the leukocyte-endothelial cell adhesion by enhancing expressions of the adhesion molecules (e.g. ICAM-1) on/in endothelial cells via the NF-κB-mediated signaling pathway [17–19]. Therefore, LPS was recruited to upregulate the level of pP65 in the nuclei. Prior to western blotting, the increasing expressions of ICAM-1 induced by LPS at the increasing concentrations (i.e. 0, 0.1, 0.5, and 1 μg/mL, respectively for 12 h) were confirmed at both protein and mRNA levels by immunofluorescence imaging and RT-PCR plus agarose gel electrophoresis, respectively (Additional file 1: Fig. S5).

**Fig. 8:**
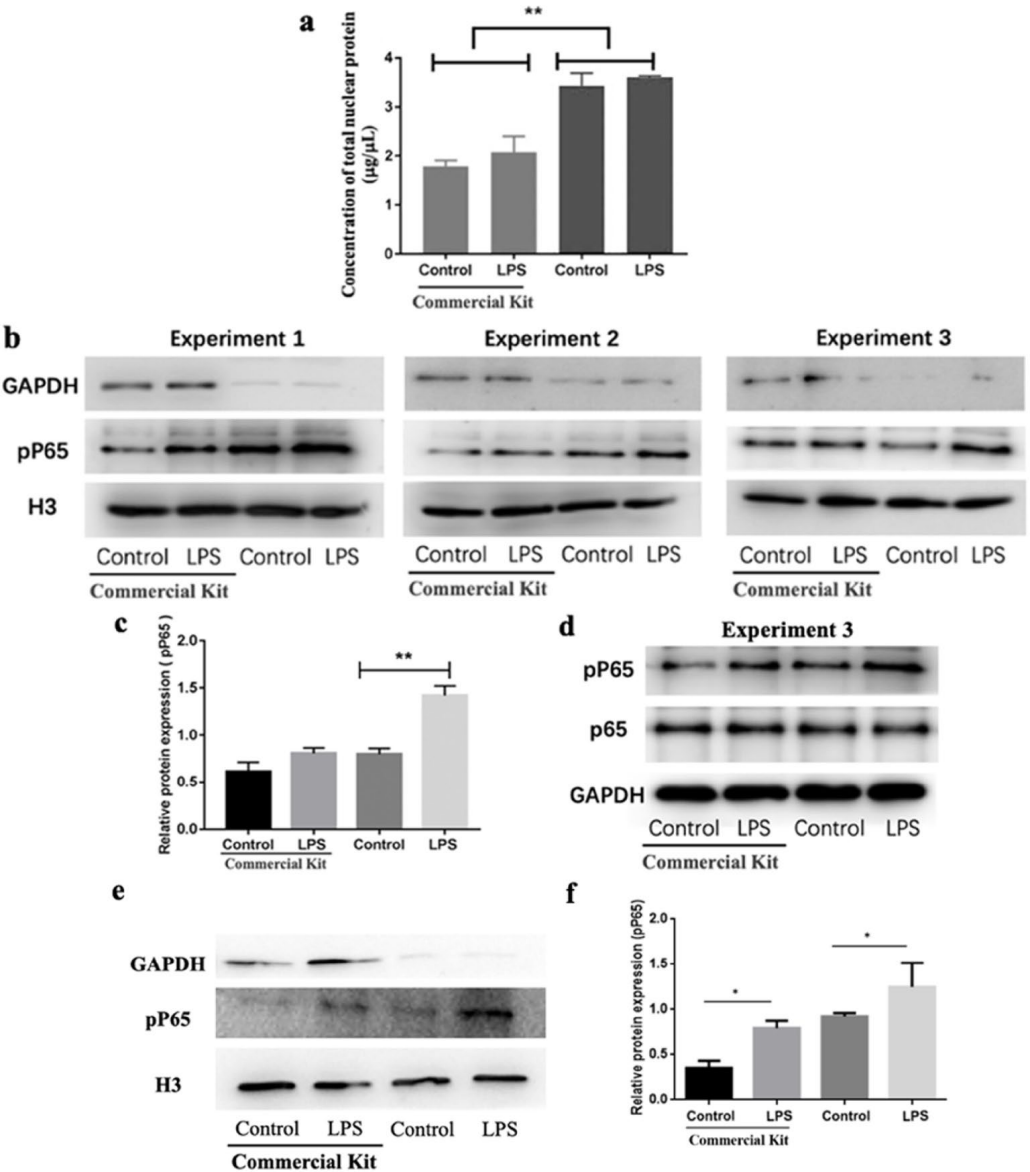
Validation of the effectiveness and sensitivity of in situ nuclear isolation for the evaluation of pP65 by western blotting. **a** Quantification of total nuclear proteins of HUVECs obtained via different methods. The final volume of the lysates is approximately 100 μL for all groups. **b** Western blot of pP65, H3 (histone H3), and GAPDH in nuclei of HUVECs from three independent experiments. **c** Quantitative analysis of the content of pP65 in nuclei of HUVECs (ratio of pP65 to H3). **d** Western blot of pP65, P65, and GAPDH in the cytoplasm of HUVECs which is collected from the supernatant after 0.1% Triton treatment in experiment 3. **e** Representative western blot of pP65, H3, and GAPDH in nuclei of HepG-2 cells. **f** Quantitative analysis of the content of pP65 in nuclei of HepG-2 cells (ratio of pP65 to H3). The LPS group represents the cells treated with 1 μg/mL LPS for 12 h before western blot. *p < 0.05; **p < 0.01 compared with the commercial kit (**a**) or with the control (**c**, **f**)

Next, the nuclei and nuclear proteins from the HUVEC cells treated with or without 1 μg/mL LPS were prepared by using our method and a commercial kit as a control, respectively. The total nuclear proteins were quantified via the traditional BCA protein assay (Fig. 8a). We found that the concentrations of total nuclear proteins obtained by our method (3.43 ± 0.37 μg/μL and 3.61 ± 0.04 μg/μL for the control and LPS groups, respectively) are significantly (almost twofold) higher than those obtained by the commercial kit in both control (1.79 ± 0.17 μg/μL) and LPS (2.08 ± 0.46 μg/μL) groups (Fig. 8a), partially reflecting a higher effectiveness of our method (strategy 1) as compared with the commercial kit.

Then, the level of the target protein (pP65) in nuclear proteins of HUVECs was evaluated via western blotting. Figure 8b shows the electrophoretic results of three experiments. It is obvious that compared with the commercial kit our method obtained relatively thicker bands of pP65 in both control and LPS groups and that the bands of pP65 in LPS group were much thicker than those in the control group (Fig. 8b). The quantitative analysis of the ratio of pP65 to histone H3 (an internal reference for nuclear proteins) shows that in our method a higher pP65 level in nuclei was detected in both control and LPS groups compared with that in the commercial kit method (Fig. 8c), confirming the observation of western blot bands and implying a higher sensitivity of our method (strategy 1) as compared with the commercial kit.

Glyceraldehyde-3-phosphate dehydrogenase (GAPDH) is an enzyme for glycolysis (a biological process converting glucose into pyruvate) which happens in the cytosol and generally is widely used as an internal reference for cytoplasmic proteins during western blotting. The GAPDH level is relatively low in the nucleus unless nuclear translocation/accumulation of GAPDH occurs following cell toxicity/apoptosis or in pathogenesis of diseases (e.g. Parkinson’s disease and Alzheimer’s disease) [20–23]. In this study, GAPDH was utilized as an internal reference for evaluating the cytoplasmic contamination of nuclear proteins. Interestingly, we found that GAPDH bands in both control and LPS groups obtained by our method were much thinner than those obtained by the commercial kit (Fig. 8b), implying that the total nuclear proteins obtained by our method (strategy 1) were barely contaminated by cytoplasmic proteins.

The level of pP65 in nuclear proteins of HepG-2 cells also was evaluated via western blot. Figure 8e and f display a representative electrophoretic result and the quantitative analysis (the ratio of pP65 to H3), respectively. The data show that compared with the commercial kit our method obtained relatively thicker bands of pP65 in both control and LPS groups and that the bands of pP65 in LPS group were much thicker than those in the control group. Figure 8e also shows that GAPDH bands in both control and LPS groups obtained by our method were much thinner than those obtained by the commercial kit, which coincides with the data on HUVECs.

The effectiveness, sensitivity, and cytoplasmic contamination of strategy 2 of our method were not tested in this study since we thought that strategy 1 (Fig. 1b) may be better than strategy 2 (Fig. 1c) because of the following disadvantages of strategy 2. For in situ isolation of nuclear proteins (strategy 2), a relatively large volume of SDS solution will be used to disrupt all nuclei adhered on the substrate with a large surface area; moreover, some nuclear proteins may stick on the large substrate surface resulting in a potential loss of some nuclear proteins (this problem can be partially addressed by washing repeatedly with a larger volume of liquid but causing further dilution of the nuclear proteins). Therefore, an extra step will be needed to concentrate the nuclear proteins by using a relatively more demanding method/equipment for strategy 2 of our method.

Taken together, our data provide evidence for the feasibility of in situ isolation of nuclei and nuclear proteins from living adherent cells (corresponding to strategies 1 and 2, respectively) and for the optimal triton concentration range (approximately 0.1–1% for ~ 10 min), optimal treatment time (< 30 min), effectiveness, sensitivity, and less cytoplasmic contamination of our method (strategy 1) as compared with a commercial nuclear protein extraction kit which is based on the isolation of nuclei from suspended cells. Our data also implies that under proper conditions (0.1–1% for < 30 min) Triton X-100 can remove the extranuclear parts of a cell while lower concentration and shorter treatment remain some extranuclear components whereas higher concentration and longer treatment may cause the release of some intranuclear proteins. However, we have to admit that the selected nuclear/cytoplasmic markers/proteins in this study could not represent most proteins and that more proteins must be tested in the future by other investigators. Moreover, further in-depth studies on strategy 2 of our method and on the comparison of our method with other commercial kits will be needed in the future. Our study provides a simple effective isolation method of nuclei or nuclear proteins with less cytoplasmic contamination from adherent cells not only for evaluation of specific proteins in the nucleus by western blotting but also for nuclear proteomic profiling and other aims. Although out of the main objective of this study, it is worth mentioning that our method (strategy 1) also has the potential of isolating cytoplasmic proteins for western blotting because our preliminary data shows that compared with the commercial kit our method obtained relatively thicker bands of cytoplasmic pP65 in both control and LPS groups (Fig. 8d).

## Supplementary Information

**Additional file 1: Fig. S1.** In situ confocal observation of the effects of digitonin (another type of detergent) on HUVECs. **Fig. S2.** Fluorescence confocal imaging visualizes the effects of Triton X-100 (0.1% for 10 min) on intranuclear contents of HepG-2 cells including chromosomes and proteins (e.g. pP65 as a representative). The HepG-2 cells have been activated by 1 μg/mL LPS prior to the Triton treatment. The chromosomes were stained with DAPI (blue), and pP65 proteins were stained with anti-pP65 antibody and AlexaFluor647-conjugated anti-IgG antibody (red). Scale bar: 20 μm. **Fig. S3.** In situ observation of the effects of gentle washes on the nuclei remaining on the substrate after Triton X-100 treatment. **Fig. S4.** Comparison between the yield of nuclear proteins (Hif-1α and pP65) isolated by our method from HUVECs and their yield from the total proteins isolated by RIPA solution from cells at the same density. **Fig. S5.** Confirmation of the LPS-induced expression of ICAM-1 in a concentration-dependent manner.

**Additional file 2: Video S1.** The time-lapse observation of the effects of successive Triton X-100 (0.1%) and SDS (0.2%) treatments on the mitochondria and nuclei of cells.

## Data Availability

The datasets used and/or analysed during the current study are available from the corresponding author on reasonable request.
