# Supplementary material for: In situ isolation of nuclei or nuclear proteins from adherent cells: a simple, effective method with less cytoplasmic contamination
Source: Biol Res. 2023 Apr 21;56:18. doi: 10.1186/s40659-023-00429-2 (PMC10120145; doi:10.1186/s40659-023-00429-2)
Supplement: Supplementary file 1 — Additional file 1: Fig. S1. In situ confocal observation of the effects of digitonin (another type of detergent) on HUVECs. Fig. S2. Fluorescence confocal imaging visualizes the effects of Triton X-100 (0.1% for 10 min) on intranuclear contents of HepG-2 cells including chromosomes and proteins (e.g. pP65 as a representative). The HepG-2 cells have been activated by 1 μg/mL LPS prior to the Triton treatment. The chromosomes were stained with DAPI (blue), and pP65 proteins were stained with anti-pP65 antibody and AlexaFluor647-conjugated anti-IgG antibody (red). Scale bar: 20 μm. Fig. S3. In situ observation of the effects of gentle washes on the nuclei remaining on the substrate after Triton X-100 treatment. Fig. S4. Comparison between the yield of nuclear proteins (Hif-1α and pP65) isolated by our method from HUVECs and their yield from the total proteins isolated by RIPA solution from cells at the same density. Fig. S5. Confirmation of the LPS-induced expression of ICAM-1 in a concentration-dependent manner. [file 40659_2023_429_MOESM1_ESM.docx]

**Supplementary Materials**

*In situ* isolation of nuclei or nuclear proteins from adherent cells: A simple, effective method with less cytoplasmic contamination

Ying Qin^1,2,#^, Yun Zhou^1,2,#^, Kun Wang^2^, Jiaxuan Gu^2^, Zhihao Xiong^2^, Wendiao Zhang^2^, and Yong Chen^1,2,*^

^1^ Jiangxi Key Laboratory for Microscale interdisciplinary Study, Institute for Advanced Study, Nanchang University, Nanchang, Jiangxi330031, China

^2^ College of Life Sciences, Nanchang University, Nanchang, Jiangxi 330031, P. R. China

# The two authors equally contributed to the study.

***** Correspondence: dr_yongchen@hotmail.com or tychen@ncu.edu.cn

**Supplementary Materials:**

**Legend to Supplementary Video 1 (Video S1)**

**Supplementary Figure 1 (Fig. S1)**

**Supplementary Figure 2 (Fig. S2)**

**Supplementary Figure 3 (Fig. S3)**

**Supplementary Figure 4 (Fig. S4)**

**Video S1. The time-lapse observation of the effects of successive Triton X-100 (0.1%) and SDS (0.2%) treatments on the mitochondria and nuclei of cells.** HUVECs were fluorescently stained for the mitochondria and nuclei with Mitotracker Red CMXRos (red) and Hoechst33342 (blue), respectively (both were purchased from Beyotime Institute of Biotechnology, Haimen, China). The replacements of Triton/SDS solutions including the removing of previous solutions and the addition of fresh solutions were performed on the stage of confocal microscope and the video also included the periods of solution replacements. Before treatments, the suspension is clear and the fluorescently stained mitochondria (red) were confined inside the clear boundary of cells with fluorescently stained nuclei (blue). After 0.1% Triton addition, the cell boundary quickly became subtle, many particles were released from the cytosol and appeared in suspension, then the red fluorescence (mitochondria) gradually disappeared, and mainly nuclei remained on the substrate. These phenomena implied the breakdown of the plasma membrane and the release of cytoplasmic contents including fluorescently stained mitochondria. On the other hand, during the whole process of Triton treatment the nuclei remained unchanged (e.g. the nuclear boundary/membrane, the particles inside the nuclei, and the blue fluorescence), implying that the cell nuclei were not disturbed by Triton. However, after 0.2% SDS treatment, the nuclear boundary rapidly blew up, the particles inside nuclei spread outward, and the blue area became larger and larger and finally disappeared), implying that SDS treatment caused the breakdown of nuclear membrane and the release of the nuclear contents. Scale bar: 20 μm.


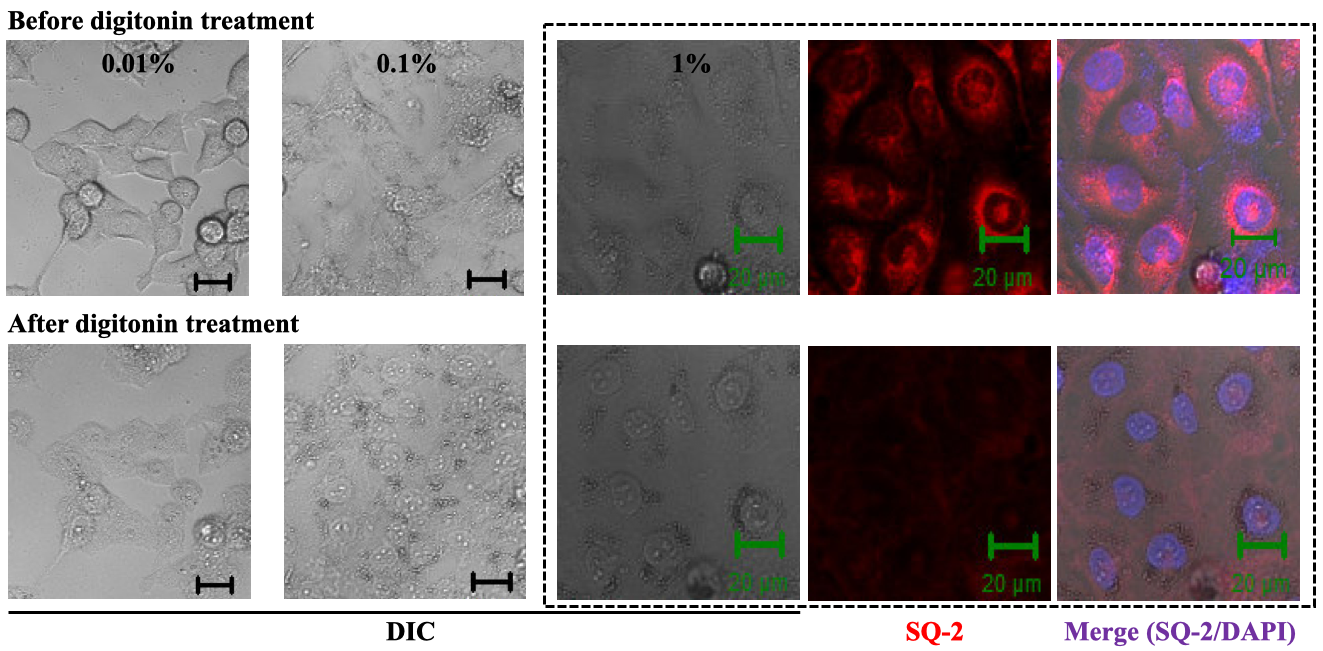


**Fig. S1. *In situ* confocal observation of the effects of digitonin (another type of detergent) on HUVECs.** Different concentrations of digitonin (0.01%, 0.1%, and 1%, respectively) were used to treat HUVECs for 10 min. Upper panels: before treatment; bottom panels: digitonin treatment for 10 min. For the 1% digitonin treatment, the cells were stained with SQ-2 (red) and DAPI (blue) for cytoplasmic and intranuclear contents of cells, respectively (as indicated by the dashed box). Scale bar: 20 μm. The data shows that digitonin even at a relatively high concentration (1%) could not completely deplete the cytoplasmic contents of cells.


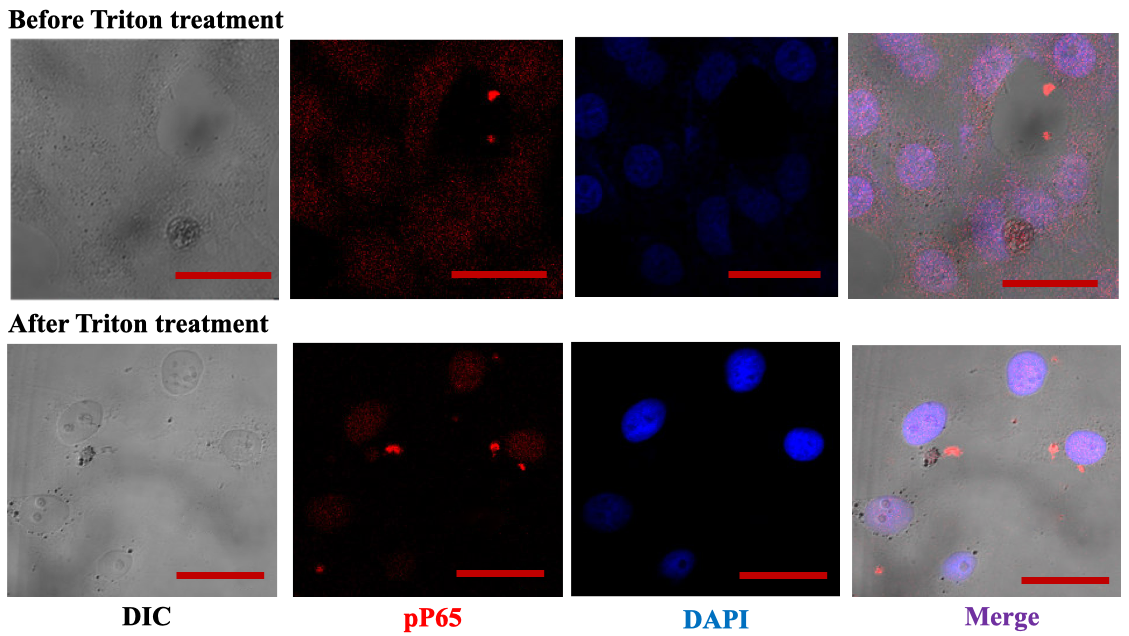


**Fig. S2.** Fluorescence confocal imaging visualizes the effects of Triton X-100 (0.1% for 10 min) on intranuclear contents of HepG-2 cells including chromosomes and proteins (e.g. pP65 as a representative). The HepG-2 cells have been activated by 1 μg/mL LPS prior to the Triton treatment. The chromosomes were stained with DAPI (blue), and pP65 proteins were stained with anti-pP65 antibody and AlexaFluor647-conjugated anti-IgG antibody (red). Scale bar: 20 μm.


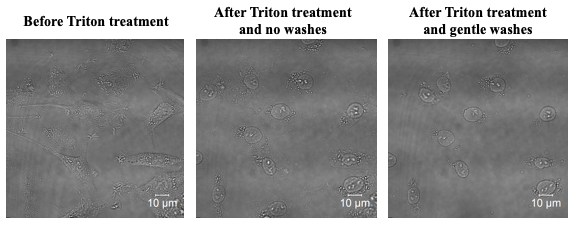


**Fig. S3. In situ observation of the effects of gentle washes on the nuclei remaining on the substrate after Triton X-100 treatment.** Left panel: the cells without triton treatment; middle panel: the remaining cell nuclei after Triton treatment; right panel: the remaining cell nuclei after Triton treatment and several gentle washes with PBS.


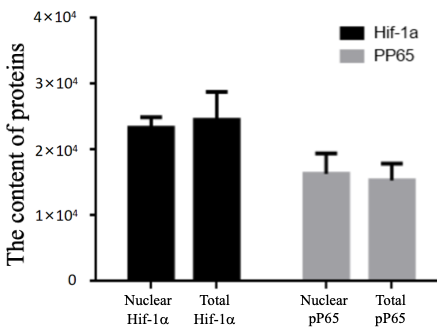


**Fig. S4. Comparison between the yield of nuclear proteins (Hif-1α and pP65) isolated by our method from HUVECs and their yield from the total proteins isolated by RIPA solution from cells at the same density.**


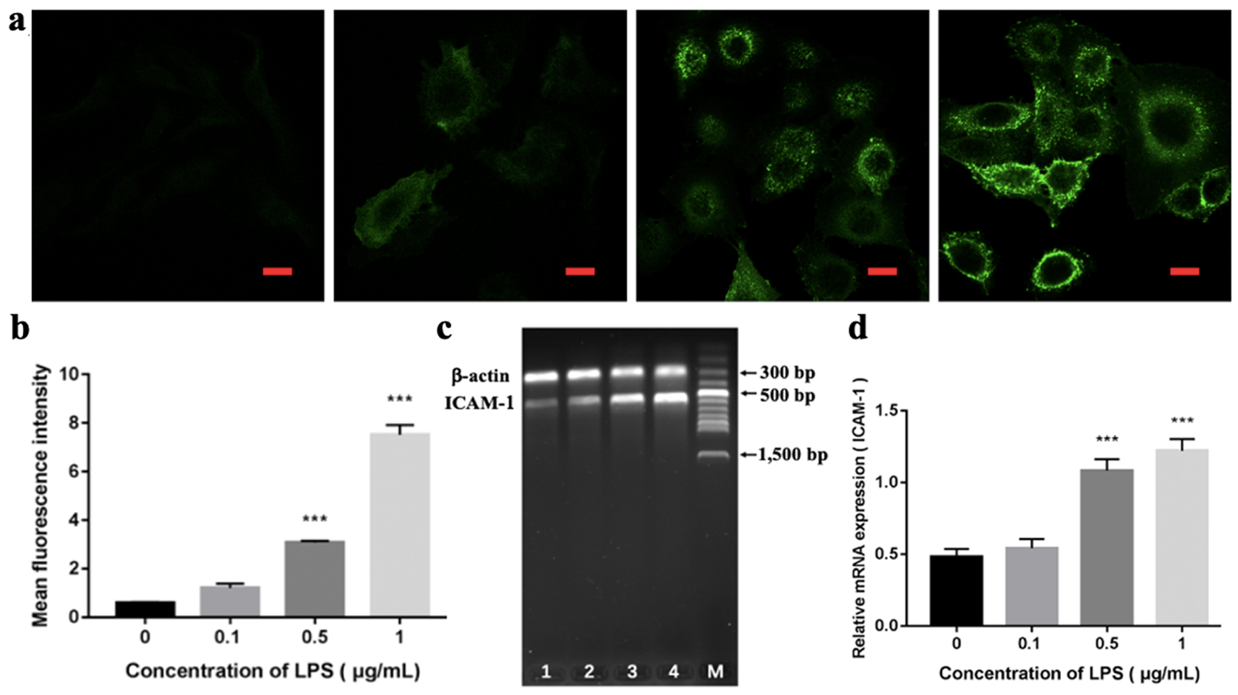


**Fig. S5. Confirmation of the LPS-induced expression of ICAM-1 in a concentration-dependent manner.** (**a**) Fluorescence images of ICAM-1 molecules on HUVECs treated with 0, 0.1, 0.5, and 1 μg/mL LPS, respectively (from left to right). Scale bars: 20 μm. (**b**) The mean fluorescence intensity (MFI) quantification. (**c**, **d**) Agarose gel electrophoresis and quantification (the ratio of ICAM-1 to β-actin) of ICAM-1 mRNA expression in the cells treated with 0, 0.1, 0.5, and 1 μg/mL LPS, respectively (lanes 1-4; lane M: DNA ladder). ***, *p* < 0.001 compared with the control.
